# The heat exposure, aging trajectories, and Alzheimer’s disease (HEAT-AD) study protocol

**DOI:** 10.1371/journal.pone.0350611

**Published:** 2026-07-09

**Authors:** Deirdre M. O’Shea, Lun-Ching Chang, Moushume Chowdhury, Diane Joyce Cook, Kevin Cresswell, Bryan D. Minor, Diana Mitsova, Madeleine Samantha Tourelle, James E. Galvin, Lilah M. Besser

**Affiliations:** 1 Department of Neurology, Comprehensive Center for Brain Health, University of Miami, Florida, United States of America; 2 Department of Mathematics and Statistics, Florida Atlantic University, Florida, United States of America; 3 School of Electrical Engineering and Computer Science, Washington State University, Washington, United States of America; 4 Department of Urban and Regional Planning, Florida Atlantic University, Florida, United States of America; Public Library of Science, UNITED KINGDOM OF GREAT BRITAIN AND NORTHERN IRELAND

## Abstract

Urban heat islands (UHIs) are areas of elevated surface temperature caused by limited vegetation and dense development. Extreme heat disproportionately affects older adults and may increase risk for Alzheimer’s disease (AD) and related dementias (ADRD), but few studies have examined whether cumulative neighborhood-level heat exposure is associated with cognitive decline or ADRD biomarkers. This study will investigate whether recent UHI exposure is associated with cognitive function; determine whether cumulative UHI exposure is associated with cognitive decline, plasma ADRD biomarkers, and brain imaging biomarkers; and evaluate whether UHI exposure is associated with ADRD risk factors, including physical inactivity, social isolation, and inflammation, and whether tree canopy mitigates heat-ADRD associations. The study includes 500 adults aged ≥50 years from the Healthy Brain Initiative cohort in South Florida. Geocoded residential addresses will be linked to satellite-derived land surface temperature data to quantify recent exposure at the baseline cognitive visit and cumulative exposure during the month before, and 1 and 5 years before baseline. Participants complete annual clinical and neuropsychological assessments, brain MRI, and blood collection for ADRD biomarkers. A sub-sample of 200 participants will wear smartwatches during two 3-week periods to capture GPS-based heat exposure, physical activity, and daily cognitive testing. Analyses will be replicated using the nationally representative Health and Retirement Study. Primary outcomes include cognitive domain scores, brain imaging measures, and preclinical AD status determined from plasma biomarkers. This study will provide comprehensive data on cumulative UHI exposure and ADRD risk by integrating cognitive assessment, neuroimaging, and biomarkers. Findings will clarify whether neighborhood heat represents a modifiable ADRD risk factor and inform urban greening and brain health promotion strategies.

## Introduction

Alzheimer’s disease and related dementias (ADRD) represent a major and growing public health challenge, currently affecting an estimated 7.2 million U.S. adults aged 65 years and older and projected to affect 13.8 million by 2060 [1]. The burden of ADRD is borne not only by people living with ADRD but also by families, caregivers, and health systems [1]. This burden is further shaped by health disparities that disproportionately affect certain populations, including economically under-resourced and medically underserved populations [2].

Although advancing age and genetic susceptibility are among the strongest predictors of ADRD [1], up to 45% of dementia cases may be attributable to potentially modifiable risk factors, such as physical inactivity, social isolation, and high low-density lipoprotein cholesterol [3]. Accordingly, prevention efforts have increasingly emphasized brain health promotion and the identification of upstream exposures that may influence neurodegenerative processes before the onset of clinical symptoms [2,3]. Identifying environmental and social determinants that shape these pathways is therefore essential for informing population-level prevention strategies [2,4].

Concurrently, extreme heat is an increasingly important public health threat. If global mean temperature rises just under 2°C, annual heat-related deaths are projected to increase by 370% by mid-century [5], and heat-related mortality among adults older than 65 years has already increased substantially relative to the 1990s [6]. Risks are greatest among older adults, people with chronic medical conditions, and those with fewer economic resources or less capacity to avoid or mitigate exposure [7–9]. Older adults and populations with fewer economic resources are more likely to experience both higher heat exposure and greater vulnerability to its physiological consequences [9,10].

Extreme heat may influence ADRD risk through multiple, interrelated pathways. During periods of extreme heat, many adults reduce outdoor physical activity, and hotter conditions may also constrain opportunities for social engagement outside the home [11,12], leading to increased social isolation (i.e., modifiable dementia risk factor) [3]. In parallel, heat exposure can trigger physiological stress responses, including activation of immune-inflammatory pathways; population studies further suggest that short-term temperature variability is associated with higher hs-CRP levels, with stronger associations observed in older adults [13,14]. Elevated inflammatory biomarkers, including hs-CRP, have in turn been associated with faster long-term cognitive decline and with greater risk of progression to dementia or Alzheimer’s disease [15,16].

Evidence directly linking heat exposure to cognitive outcomes is emerging but remains limited. Experimental studies indicate that passive or severe heat stress can acutely impair aspects of cognition, including short-term or working memory, inhibitory control, and executive function [17–19]. Longitudinal population-based studies further suggest that greater exposure to extreme heat is associated with worse cognitive performance, faster cognitive decline, and higher risk of cognitive impairment over time [8,20,21]. Findings from some studies have suggested that these associations may be stronger in populations experiencing health disparities exposed to greater environmental burden. For instance, in a U.S. cohort, higher cumulative exposure to extreme heat was associated with faster cognitive decline among Black older adults and residents of poorer neighborhoods, while in another cohort, heatwave-related cognitive risk was amplified in settings with greater air pollution and less green space [8,21]. Collectively, these findings support the possibility that chronic or repeated heat exposure may contribute to lasting cognitive vulnerability, particularly among populations facing economic challenges and health disparities.

Despite emerging evidence linking heat exposure to adverse cognitive outcomes, important limitations remain in the existing literature. Most prior studies have characterized heat exposure using ambient air temperature or heat-wave definitions, which are useful for capturing regional weather extremes, but do not capture fine-scale, within-city variation in chronic heat burden [22,23]. In contrast, surface urban heat islands (hereafter denoted as UHIs) are localized areas where land surface temperatures are elevated because dense built environments, extensive impervious surfaces, and limited vegetation absorb and retain heat more than surrounding less-developed areas [24,25]. UHI exposure can be measured using satellite-derived land surface temperature to provide a more spatially resolved indicator of neighborhood heat burden than ambient air temperature alone [25]. This distinction is important because, across U.S. cities, summer daytime UHI intensity is disproportionately higher in neighborhoods with lower economic resources and higher proportions of minoritized residents [26]. UHIs are also shaped in part by modifiable built-environment features, including tree canopy, land cover, and other heat-mitigating infrastructure, making them potentially responsive to community-level interventions such as urban greening and tree planting [24,27]. Examining neighborhood-level UHI exposure may therefore improve detection of environmentally patterned disparities in chronic heat burden and identify intervention targets that are less visible when heat is measured only with ambient air temperature or episodic heat-wave metrics [22,26].

Second, most epidemiologic studies linking heat exposure to cognition have focused on global cognitive performance or incident cognitive impairment [8,20,21,23], limiting insight into whether heat exposure is associated with specific vulnerabilities in memory, executive functioning, or other cognitive domains that may be more informative for understanding early neurodegenerative risk. Third, little is known about whether chronic heat exposure is associated with structural brain changes or biological markers of ADRD, even though such measures may detect neurodegenerative processes years before clinically observable impairment, which may provide a more sensitive test of whether heat exposure is related to underlying disease mechanisms [28].

Another limitation of prior work is its predominant focus on short-term or acute heat exposure [23]. Although acute effects are informative, cumulative exposure to elevated temperatures over months to years may be more relevant for understanding chronic disease processes, particularly in the context of ongoing increasing global temperatures and increasing heat exposure [5,8]. Repeated exposure to urban heat may compound behavioral disruption, inflammatory activation, and physiological strain, thereby contributing to accelerated cognitive aging over time [13]. Longitudinal assessments of cumulative heat exposure are therefore needed to evaluate potential dose-response relationships and to strengthen causal inference [8,23].

Taken together, existing evidence suggests that chronic heat exposure could contribute to dementia risk through converging behavioral and biological pathways [8,12,13]. However, this hypothesis has not been tested adequately using neighborhood-level heat metrics together with Alzheimer’s disease–relevant outcomes [23]. UHIs represent a persistent, spatially patterned environmental exposure that is unequally distributed across communities and shaped by modifiable built-environment features, including vegetation and other land-cover characteristics [24,26]. These characteristics make surface UHIs a particularly relevant exposure for investigating whether chronic heat burden contributes to cognitive and brain-health disparities over time.

Examining UHI exposure in relation to cognitive performance, structural brain changes, and Alzheimer’s disease biomarkers may help clarify whether environmental heat functions as a chronic risk factor for neurodegeneration rather than solely as an acute cognitive stressor. By integrating neighborhood-level heat exposure data with comprehensive neuropsychological assessment, structural neuroimaging, and plasma Alzheimer’s disease biomarkers, the present study will test whether greater cumulative exposure to surface UHI environments is associated with poorer cognitive performance, neuroimaging markers of neurodegeneration, and less favorable biomarker profiles in older adults. We will also examine whether these associations are mediated by physical inactivity, social isolation, and systemic inflammation, and whether neighborhood tree canopy coverage may mitigate urban heat exposure and be associated with more favorable cognitive and brain-health outcomes.

If associations are confirmed, findings would support specific interventions including municipal tree planting programs in communities at elevated heat risk, integration of cognitive health protection into urban plans and policies, and targeted cooling strategies for older adults in communities at elevated heat risk. Importantly, UHIs reflect modifiable characteristics of the built environment, enabling an opportunity for population-level intervention that does not depend solely on individual behavior change.

## Materials and methods

### Study setting

The HEAT-AD study is conducted in the tri-county region of Palm Beach, Broward, and Miami-Dade counties in South Florida. Based on 2024 U.S. Census Bureau county estimates [29], the region includes approximately 6.46 million residents and is characterized by substantial racial, ethnic, economic, and age diversity. Weighted tri-county estimates indicate that 48.0% of residents identify as Hispanic or Latino, 21.6% as Black or African American alone, 12.8% are living in poverty, and 19.7% are aged 65 years or older [30–32]. South Florida experiences recurrent periods of extreme heat. Official county resources identify substantial heat burden across the region: Broward County reports an average of 74 days above 88°F air temperatures per year, while Miami-Dade County reports that the number of days above 90°F has increased from 84 to 133 days per year since 1970. Palm Beach County also reports increasing extreme heat exposure and worsening UHI conditions across the county [31,33]. The region’s urbanized built environment and spatial variation in tree canopy and shade contribute to substantial neighborhood-level differences in heat exposure. These characteristics position South Florida as a well-suited setting for investigating associations between UHI exposure and cognitive health in older adults.

### Study aims, design, and overview

The HEAT-AD study is a longitudinal observational study that integrates secondary data from ongoing cohort studies with novel environmental exposure linkage and prospective wearable-based monitoring. The study leverages three complementary data sources: (1) the Healthy Brain Initiative (HBI), an ongoing longitudinal cohort study of brain aging and ADRD in South Florida (n = 500 participants aged ≥50 years) [34]; (2) a HBI-nested smartwatch study involving prospective monitoring of mobility, heat exposure, and cognitive function over two 3-week periods (n = 200 participants); and (3) the Health and Retirement Study (HRS), a nationally representative longitudinal study of aging (~9,000 participants aged ≥50 years) [35], for external validation.

#### Conceptual framework.

The study is guided by a framework in which urban heat island exposure is conceptualized as a chronic environmental stressor that may contribute to ADRD risk through both direct and indirect pathways. We hypothesize that cumulative exposure to elevated neighborhood-level land surface temperatures is associated with adverse cognitive, neuroimaging, and biomarker outcomes. We further hypothesize that these associations may be mediated by reduced physical activity, greater social isolation, and systemic inflammation, and that neighborhood tree canopy may mitigate heat exposure and thereby reduce heat-related ADRD risk.

The study is organized around three aims: 1) Investigate whether recent UHI exposure is associated with concurrent cognitive function, 2) determine whether cumulative UHI exposure is associated with longitudinal cognitive decline, plasma Alzheimer’s disease biomarkers, and structural MRI biomarkers and 3) to evaluate whether UHI exposure is associated with ADRD risk factors, including physical inactivity, social isolation, and inflammation, and whether neighborhood tree canopy is associated with lower heat exposure and more favorable ADRD-related outcomes.

### Participants and eligibility

#### HBI overall sample.

The primary analytic sample will be drawn from the HBI, an ongoing longitudinal cohort study of cognitive aging and ADRD conducted at the University of Miami [34]. At baseline, participants complete in-person assessments including informed consent, demographic and medical history questionnaires, detailed neuropsychological testing, functional assessments, blood collection, and structural MRI. Annual follow-up visits are essentially the same as the baseline and include repeat cognitive and functional assessments, with MRI obtained biennially.

HBI eligibility includes community-dwelling adults aged 50 years or older residing in Palm Beach, Broward, or Miami-Dade counties; English or Spanish speakers; Clinical Dementia Rating (CDR) [36] score ≤1 (i.e., normal cognition through mild dementia); availability of a study partner; medical eligibility for MRI; and ability to complete study procedures. Exclusion criteria include moderate or severe dementia (CDR ≥ 2), medical conditions precluding safe MRI, psychiatric or medical conditions that would substantially interfere with participation, and inability or unwillingness to provide informed consent. All currently enrolled HBI participants with geocoded residential addresses will be eligible for the present geospatial analyses. As of February 2026, the HBI cohort includes approximately 500 participants. Additional details about the HBI protocol are provided elsewhere [34].

#### HBI smartwatch sub-sample.

A nested sub-sample of HBI participants will be recruited for the smartwatch sub-study. Eligibility criteria include an HBI annual visit scheduled during the warmer months (May–October), willingness and ability to wear a smartwatch for two separate 3-week monitoring periods, willingness to maintain usual daily activities while wearing the device, and ability to charge the device nightly and follow basic care instructions. Individuals with severe skin conditions or allergies that would be aggravated by device wear will be excluded. To achieve the target of 200 participants completing both monitoring periods, up to 225 participants will be enrolled initially to account for attrition.

#### HRS validation sample.

The HRS will serve as the external validation sample. HRS is a nationally representative, longitudinal biennial panel study of U.S. adults aged 50 years and older [35]. For the present study, secondary analyses will be restricted to HRS participants with at least two cognitive assessments, available geocoded residential data linkable to environmental exposures, and residence within the contiguous United States. The anticipated analytic sample is approximately 9,000 participants. Detailed information on HRS design and recruitment has been published elsewhere [35,37].

### Recruitment and sample selection

#### HBI overall sample.

The HBI cohort is recruited from the tri-county South Florida region (i.e., Miami Dade, Broward, Palm Beach) using community-based strategies, including outreach events, partnerships with local organizations, clinician referrals, advertisements, and word-of-mouth referrals. Recruitment materials are available in English and Spanish, and the study team includes bilingual coordinators and outreach staff. Annual study visits are conducted at the University of Miami Comprehensive Center for Brain Health in Boca Raton, Florida. Transportation support and scheduling flexibility are provided to reduce barriers to participation.

#### HBI smartwatch sub-sample.

Participants for smartwatch sub-study will be recruited from HBI participants who have previously consented to be contacted about additional research opportunities. Recruitment will occur during HBI visits and through email and telephone contact. Participants will receive instructions on device use, troubleshooting support, and check-in calls during each monitoring period.

#### HRS validation sample.

No direct recruitment will be conducted for HRS, as HRS analyses will use existing secondary data.

### Consent and ethical approvals

#### HBI overall sample.

As part of the HBI protocol, participants provided written informed consent at enrollment using IRB-approved consent forms available in English and Spanish.

#### HBI smartwatch sub-sample.

For the secondary analysis portion of the HEAT-AD study, the University of Miami Institutional Review Board granted a waiver of additional informed consent (IRB protocol #20250599, approved June 17, 2025), as these analyses fall within the scope of the original consent and involve no additional participant contact or procedures.

Participants in the smartwatch sub-study will complete an electronic informed consent process through the University of Miami’s secure REDCap online database, with study staff available to answer questions and provide in-person consent support if preferred.

#### HRS validation sample.

Participants provided consent through standard HRS procedures at the University of Michigan, including authorization for secondary analysis by approved investigators. Restricted HRS geocoded data will be accessed in accordance with the applicable Restricted Data Use Agreement.

### Participant incentives

*HBI overall sample.* HBI participants receive compensation according to the parent HBI protocol for annual study visits, and study partners who accompany participants also receive compensation. No additional incentives will be provided for the HBI geospatial analyses because these involve only secondary analysis of existing data.

*HBI smartwatch sub-sample.* Participants in the smartwatch sub-study will receive a $100 gift card after each completed 3-week monitoring period, for a total of $200 if both periods are completed.

*HRS validation sample.* No incentives will be provided for HRS analyses because these involve secondary analysis without participant contact.

### Study procedures

*HBI overall sample.* Analyses involving the full HBI sample will use existing data collected as part of the standardized HBI protocol.

*HBI smartwatch sub-sample.* Participants enrolled in the smartwatch sub-study will complete two 3-week periods of wearable-based monitoring spaced approximately one year apart during the warmer months (May–October). Prior to the first smartwatch monitoring period, participants will receive in-person training on how to use the watch. They will be instructed to wear the smartwatch during waking hours, charge it nightly, and maintain their usual daily activities during monitoring, and will receive regular reminders to improve wear time compliance. No behavioral intervention will be introduced.

*HRS validation sample.* External validation analyses in HRS will use existing secondary data only, with no new participant contact or data collection**.**

### Measures for HBI overall sample (see Table 1)

*Geocoding.* Residential address information is collected from HBI participants at each study visit. Participants report the duration of residence at their current address and provide prior residential addresses and corresponding dates for up to five years before the baseline visit. All addresses will be geocoded to latitude-longitude coordinates following standardized data cleaning procedures to ensure accurate address matching. Geocoded locations will be linked to U.S. Census tracts to characterize neighborhood-level environmental exposures while accounting for residential mobility. Neighborhood measures will be derived using ArcGIS Pro (version 3.6 and later; Esri).

**Table 1:** Key HBI measures.

Variable		Source	Time period calculated
Heat	Neighborhood urban heat island (LST z-score)^a^	Satellite imagery	Last 30 days, and for 1 and 5 years prior to baseline visit (T)*
	Mean daily minutes exposed to urban heat islands (LST > 0.5 SD above mean)	Smartwatch GPS + satellite imagery	S_1_ and S_2_
Cognitive domains	Executive function, episodic memory, and global cognition	Cognitive testing	Annual HBI visit
	Working memory z-score (i.e., n-back)**	Smartwatch testing	S_1_ and S_2_
MRI	Hippocampal volume, total cortical volume in AD regions of interest (ROI), white matter hyperintensity volume (WMH)	Structural MRI	Baseline visit (T) and every two years after
Plasma AD biomarkers	Amyloid Probability Score (APS2), based on (Aβ42/40) and p-tau 217/np-tau 217 ratios	Plasma	Annual HBI visit
Neighborhood tree canopy	Neighborhood % tree canopy cover (overall, for streets, for sidewalks)^a^	Satellite imagery and LiDAR	Baseline visit (T) and S_1_
Physical activity	Quick Physical Activity Rating (QPAR)	Questionnaire	Baseline visit (T) and S_1_
Social isolation	Social isolation score	Questionnaire	Baseline visit (T) and S_1_
Inflammation	High-sensitivity CRP (hs-CRP) Lipoprotein A (lp(A))	Blood collection	Baseline visit (T) and S_1_

LST = land surface temperature; EMA: Ecological Momentary Assessment; GPS = Global Positioning System; ^a^ at Census tract level; * accounting for changes in exposure due to residential moves; ** a measure of executive function

**Fig 1:**
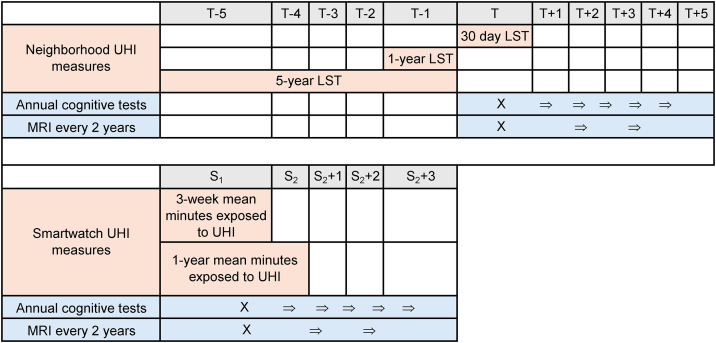
Timing of HBI UHI exposures and cognitive and MRI outcomes. The upper panel shows timing for neighborhood-level urban heat island (UHI) exposure measures derived from land surface temperature (LST) relative to the baseline HBI visit (T). Recent exposure is defined as the mean LST during the 30 days before T, and cumulative exposure is defined as mean LST during the 1-year and 5-year periods before T. Cognitive assessments are conducted annually beginning at T, and MRI is conducted at T and every two years thereafter. The lower panel shows timing for the smartwatch sub-study. Smartwatch-based UHI exposure is summarized as mean minutes exposed to UHI conditions during the first 3-week monitoring period (S1) and as the 1-year mean across the first and second smartwatch monitoring periods (S1 and S2). X indicates the index cognitive or MRI assessment, and arrows indicate follow-up assessments. Abbreviations: HBI, Healthy Brain Initiative; LST, land surface temperature; MRI, magnetic resonance imaging; S1, first smartwatch monitoring period; S2, second smartwatch monitoring period; T, baseline HBI visit; UHI, urban heat island.

*Timing of measures.* **Fig 1** depicts the timing of the UHI exposures and cognitive and MRI outcome measures for analyses involving the overall HBI sample and the smartwatch sub-sample.

**Fig 2:**
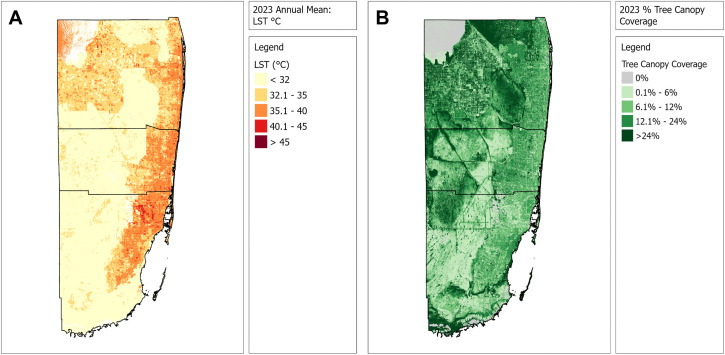
Spatial distribution of annual mean land surface temperature and tree canopy coverage in 2023. Two-panel map showing the spatial distribution of **(A)** 2023 annual mean land surface temperature (LST; °C) and **(B)** percent tree canopy coverage across the study area. Maps were generated by the authors using NASA VIIRS Collection 2 VNP21A1D daytime land surface temperature (LST) data (1 km spatial resolution), (provided by the U.S. Geological Survey (USGS) and tree canopy data from the U.S. Department of Agriculture Forest Service (USDA Forest Service). No proprietary basemap or copyrighted background imagery was used. LST values are classified as <32, 32.1–35, 35.1–40, 40.1–45, and >45 °C. Tree canopy coverage is classified as 0%, 0.1–6%, 6.1–12%, 12.1–24%, and >24%. Abbreviations: LST, land surface temperature.

*Urban Heat Island Exposure.* Neighborhood UHI exposure will be quantified using land surface temperature (LST) data derived from the NASA VIIRS Collection 2 VNP21A1D product (1km spatial resolution), available from 2012 onward [25]. Mean LST values (Fig. 2, Panel A, example) will be calculated for multiple exposure windows, including the 30 days, 1 year, and 5 years prior to each HBI visit, as well as during smartwatch monitoring periods when applicable. Daily VIIRS observations will be quality screened using the product quality assurance flags to exclude poor-quality retrievals and pixels flagged as cloudy before generating temporal composites within each exposure window.

(**Fig 2** Using zonal statistics within ArcGIS Pro [38], descriptive statistics will be calculated for LST values at both the Census tract level and the surrounding core-based statistical area (CBSA). Standardized LST z-scores will be calculated by subtracting the CBSA-level mean LST from the participant’s Census tract-level mean LST and dividing by the CBSA-level standard deviation, with higher scores indicating relatively hotter neighborhood environments [39]. As a secondary heat exposure measure, the number of extreme heat days will be determined using data from the CDC National Environmental Public Health Tracking Network [40] and linked to participants’ Census tracts over the same exposure windows. Satellite-derived land surface temperature (LST) estimates may be affected by cloud cover, particularly in subtropical regions such as South Florida, where frequent cloud contamination can limit data availability during certain time periods. To address this, standard cloud masking procedures will be applied, and LST values will be derived using temporal compositing across multiple scenes within each exposure window. If persistent data gaps remain, particularly for shorter aggregation periods (e.g., 30-day or monthly means), alternative LST estimation approaches or supplementary datasets with varying temporal resolution will be incorporated to improve spatial and temporal completeness at the neighborhood scale.

*Neighborhood Tree Canopy.* Neighborhood tree canopy coverage (**Fig 2** Panel B, example), which includes branches, leaves, and stems covering the ground as viewed from above, will be quantified using the USDA Forest Service’s Tree Canopy dataset [41]. Our primary measure will be the percentage of tree canopy coverage for participants’ residential Census tract locations. The LiDAR Analyst (Light Detection and Ranging) extension for ArcGIS Pro enables the extraction of vegetation structural metrics, such as tree height, canopy cover, and stem diameter, from raw LiDAR point cloud data. Exploratory measures will be calculated, specifically, percentage of neighborhood streets with tree canopy cover within 6m, and percentage of neighborhood sidewalks with trees canopy coverage within 6m. To accomplish this, LiDAR will be downloaded from the National Oceanic and Atmospheric Administration (NOAA) [42] to determine locations of trees in the Census tracts where participants reside. These tree locations will be overlaid on a street network map to determine the percentage of streets and sidewalks (calculated separately) with nearby tree canopy coverage (within 6m), and these values will be calculated for each Census tract. Additional exploratory measures that can be derived using LiDAR will also include total number of neighborhood trees and neighborhood canopy area (determined from canopy diameter). These measures will be used to examine tree canopy as both a predictor of lower LST exposure and as a moderator of associations between heat exposure and ADRD outcomes.

*Neighborhood Socioeconomic Status.* Neighborhood socioeconomic context will be indexed using the Area Deprivation Index (ADI), obtained from the University of Wisconsin Neighborhood Atlas. The ADI is a census block group-level measure derived from American Community Survey indicators of income, education, employment, and housing, with higher scores indicating lower neighborhood resources (range: 1–100) [43]. ADI values will be assigned based on a participant’s residential location and summarized at the Census tract level for the baseline visit, smartwatch monitoring periods S_1_ and S_2_, and as mean exposures over the 1-year and 5-year periods preceding baseline. Mean ADI across S_1_ and S_2_ will also be calculated to capture cumulative neighborhood resources during the monitoring period.

*Neighborhood air pollution (PM*_*2.5*_*).* Ambient fine particulate matter (PM_2.5_; particles <2.5 µm in aerodynamic diameter) has been associated with increased ADRD risk [44–46]. PM_2.5_ concentrations will be estimated using EPA monitoring stations located across Palm Beach, Broward, and Miami-Dade counties. Established deterministic and probabilistic interpolation methods in ArcGIS Pro will be used to spatially interpolate values between monitors for participant residential locations. Variograms will be used to cross-validate predicted values, identify inconsistencies, and reduce prediction error. Mean PM_2.5_ concentrations will be calculated for the 30 days prior to each HBI visit, the 1- and 5-year periods prior to each visit, and across each smartwatch monitoring period (S_1_ and S_2_), reflecting cumulative PM_2.5_ exposure. For the three South Florida counties from January to December 2023, estimated mean (SD) PM_2.5_ concentrations were 7.7 (3.3) µg/m³ in Palm Beach County (range: 0.0–30.5), 8.2 (3.5) µg/m³ in Broward County (range: 0.0–36.9), and 8.1 (3.3) µg/m³ in Miami-Dade County (range: 0.0–34.4). These values approach national standards for unhealthy PM_2.5_ exposure (≥9 µg/m³). PM_2.5_ will be evaluated as a potential confounder in analyses of the association between UHI exposure and ADRD-related outcomes.

*Cognitive assessment measures.* HBI participants complete a comprehensive neuropsychological assessment battery at baseline and each annual follow-up visit, as described previously in the HBI cohort protocol [34]. The battery incorporates the National Alzheimer’s Coordinating Center Uniform Data Set Version 4.0 (NACC UDS v4.0) neuropsychological battery [47], including Craft Story 21 Immediate and Delayed Verbatim and Paraphrase Recall, Benson Complex Figure Copy and Recall, Number Span Forward and Backward, Category Fluency (Animals, Vegetables), Letter Fluency for “F” and “L”, Trail Making Test Parts A and B, and the Multilingual Naming Test, as well as the Montreal Cognitive Assessment (MoCA) and supplementary measures used in HBI, including the Hopkins Verbal Learning Test–Revised (HVLT-R) [48], Number Symbol Coding Task (NSCT) [49], and the Noise Pareidolia Task [50,51].

*Cognitive outcomes.* In prior work using the baseline HBI sample, confirmatory factor analysis of 16 cognitive indicators identified latent factors representing episodic memory, executive function, language, and global cognition. Episodic memory was indicated by HVLT-R total learning and delayed recall, Craft Story 21 immediate and delayed verbatim recall, Benson Complex Figure recall, and MoCA delayed recall. Executive function was indicated by Trail Making Test Parts A and B time to completion scores, reverse-scored so that higher values reflected better performance, NSCT total score, Number Span Forward and Backward total correct trials, and selected MoCA attention items. Language was indicated by Category Fluency (Animals) total correct, MINT total score, MoCA phonemic fluency, and MoCA sentence repetition. A latent global cognitive factor was specified across all 16 indicators. Visuospatial performance did not fit the latent structure adequately and was therefore operationalized separately as a composite derived from Benson Complex Figure Copy, selected MoCA visuospatial items, and Noise Pareidolia performance. In the present study, these previously derived latent and composite outcomes will be used. As additional participants complete baseline and annual follow-up assessments, factor scores will be generated using the same previously established measurement model.

The primary cognitive outcome for cross-sectional analyses is the executive function factor score, given that executive function is the domain most consistently associated with acute or recent extreme heat exposure in prior studies [19,52,53].The primary cognitive outcomes for longitudinal analyses are change in global cognition and change in the episodic memory factor score; global cognition has been associated with cumulative heat exposure in longitudinal observational work [8,54,55], and episodic memory is the domain most characteristically affected in early AD [56]. All remaining domain scores will be examined as secondary outcomes.

*Structural Magnetic Resonance Imaging Outcomes.* Structural brain MRI data are acquired as part of the parent HBI study protocol. Briefly, participants undergo scanning on a GE 3 Tesla 750W scanner at baseline and every two years using sequences modified from the Alzheimer’s Disease Neuroimaging Initiative 3 (ADNI-3) canonical protocol [57]. The acquisition includes high-resolution three-dimensional MPRAGE images with axial and coronal reconstructions, axial 3D T2 FLAIR, 2D T2* sequences, diffusion tensor imaging, and resting-state BOLD sequences. The present study will use T1-weighted MPRAGE and FLAIR sequences for the MRI outcomes described below.

Cortical and subcortical volumes are extracted using the Combinostics cNeuro® cMRI™ suite (Tampere, Finland), an FDA-cleared AI-driven quantitative neuroimaging pipeline with automated segmentation accuracy comparable to manual methods showing an intraclass correlation coefficients of 0.83–0.84 for hippocampal segmentation, dice similarity index of 0.88 and demonstrated superiority over visual rating scales for detecting cortical atrophy in cognitively impaired populations [58,59]. Three volumetric outcomes, normalized by intracranial volume (ICV), will be examined in the present study: 1) total left and right hippocampal volume (cm³); 2) total WMH volume (cm³) from FLAIR sequences (log-transformed if substantially skewed) and 3) total gray matter volume (cm³) across four AD-relevant cortical regions of interest including entorhinal cortex, fusiform gyrus, inferior temporal gyrus, and middle temporal gyrus, selected for their well-established vulnerability to early AD-related neurodegeneration [60,61].

*Plasma Biomarkers of Alzheimer’s Disease Outcomes.* Blood samples are collected from HBI participants at baseline and annual follow-up visits using standardized phlebotomy procedures. Blood is collected in EDTA tubes and plasma is isolated via centrifugation, aliquoted, and stored at −80°C, within 1 hour of blood collection. Plasma samples are processed by C2N Diagnostics (St. Louis, MO, USA) using liquid chromatography-tandem mass spectrometry (LC-MS/MS). The primary outcome for cognitively unimpaired HBI participants (77.2% of the sample) will be the Amyloid Probability Score 2 (APS2), generated by the PrecivityAD2 test. APS2 is a 0–100 composite score derived from a proprietary algorithm integrating the plasma Aβ42/40 ratio and %p-tau217 (the percentage of tau phosphorylated at threonine 217 relative to non-phosphorylated tau217), with higher scores indicating greater likelihood of brain amyloid positivity. In the clinical validation study, APS2 demonstrated 88% sensitivity and 89% specificity relative to amyloid PET; under the current reporting framework, scores of 48–100 are interpreted as positive and 0–47 as negative [62]. Approximately 17.5% of cognitively unimpaired HBI participants currently meet this threshold. Exploratory analyses will additionally examine in-house plasma measures of glial fibrillary acidic protein (GFAP), a marker of astrogliosis/astrocytic activation, and neurofilament light chain (NfL), a marker of neuroaxonal injury [63]. These biomarkers are quantified in the HBI laboratory using the Quanterix Simoa SR-X platform (Quanterix, Billerica, MA), an ultrasensitive immunoassay-based system for blood biomarker measurement [64].

*Potential mediators.* Several variables will be examined as potential mediators of associations between heat exposure and cognitive outcomes in HBI. Physical activity will be assessed using the Quick Physical Activity Rating (QPAR) [65], which measures the intensity, frequency, and duration of 10 activity categories reported over the previous 4 weeks, with higher scores indicating greater activity. Social isolation will be assessed using the Social Isolation Score subscale score from the Resilience Index [66], which captures current social activity, socialization, and engagement, with lower scores indicating greater isolation. Blood-based inflammatory and vascular risk markers will include lipoprotein(a) [Lp(a)] and high-sensitivity C-reactive protein (hs-CRP), assayed for HBI participants through Cleveland HeartLab.

*Covariates.* Covariates for analyses using HBI data will include self-reported age, sex, marital status, socioeconomic position, baseline cognitive status, and relevant medical and psychiatric comorbidities. Socioeconomic position will be indexed using the Hollingshead Index of Social Position [67], and baseline cognitive status will be represented by the global Clinical Dementia Rating Scale (CDR) [36] score categorized as 0 versus 0.5/1. Medical covariates will include cardiovascular disease, cerebrovascular disease, diabetes, and hypertension. Diabetes will be defined by elevated HbA1c and/or diabetes medication use, and hypertension by measured blood pressure at the study visit and/or antihypertensive medication use. Depression and anxiety symptoms will be assessed using the Hospital and Anxiety Depression Scale (HADS) [68]. Additional candidate covariates may include seasonality and data on precipitation and humidity, downloaded from local and national data sources (e.g., National Centers for Environmental Information) [69], other measures of physical and leisure activity, described elsewhere [34].

### Measures for HBI smartwatch sub-study

Smartwatches will collect GPS locations for each 3-week monitoring period. Additionally, each evening between 6:30 pm and 8:30 pm, the smartwatch will prompt participants to complete three Ecological Momentary Assessments (EMA) and two cognitive tasks.

*Smartwatch geolocation.* Smartwatch data collection and feature extraction will be conducted by the Washington State University team using their validated Apple Watch application [70]**.** Continuous sensor readings, including accelerometer, gyroscope, and heart rate data, will be sampled at 10 Hz. GPS coordinates (latitude and longitude) will be collected every five minutes, or more frequently when accelerometer values indicate a likely change in location. Participants’ home location will be defined as the location visited most often between 2:00 and 9:00 AM. OpenStreetMap and Florida’s Geographic Data Library will be used to reverse geocode GPS coordinates and classify location types (e.g., road, home, hospital, church, park), from which time spent inside and outside the home will be derived.

*Smartwatch heat measures.* Individual-level urban heat island exposure will be estimated by linking daily GPS coordinates to the LST maps described above. The primary smartwatch-based heat exposure measure will be mean daily minutes spent in locations where LST exceeds 0.5 SD above the regional mean, calculated separately for the baseline 3-week monitoring period (S_1_) and as a one-year mean across both monitoring periods (S_1_ and S_2_). GPS coordinates will also be linked to local weather station data to derive mean outdoor air temperature and number of extreme heat days for each monitoring window. Exploratory measures will include the daily percentage of time and total minutes spent walking in UHI environments.

*Smartwatch Cognitive Assessments.* Each evening, participants complete two brief cognitive tasks. The first is a validated mobile implementation of the n-back task [70,71], a measure of working memory in which participants monitor a sequential series of stimuli and indicate when the current stimulus matches one presented one position earlier (1-back condition), administered for approximately 45 seconds. Daily n-back working memory scores collected during the baseline smartwatch period (S_1_) will serve as the primary cross-sectional smartwatch cognitive outcome. The second is an adapted mobile version of the Deary-Liewald Reaction Time Task [72] (“reaction test,” < 2 minutes), a measure of processing speed in which participants view a blank screen and tap as rapidly as possible each time an “X” appears at unpredictable intervals across multiple trials; reaction time is sensitive to thermal strain at relatively low heat loads [17]. The primary outcome metrics are score mean and slope for the n-back task and median response latency (milliseconds) for the reaction time task.

*Smartwatch ecological momentary assessment (EMA).* Participants will receive a brief EMA survey via the smartwatch application each evening. The survey includes three items assessing heat-related behavior and daily social context: (1) whether the participant avoided going outside or changed activities because of hot weather (yes/no); (2) the participant’s current activity (e.g., errands, meal, relax, exercise); and (3) how much time the participant spent with other people that day, rated on a 5-point Likert scale from none to quite a bit. These EMA items will be used to characterize heat avoidance behavior and social contact in daily life.

### Measures for HRS replication sample

#### Neighborhood UHI exposure.

To parallel the HBI analyses, neighborhood UHI exposure in HRS will be characterized using Census tract-level land surface temperature (LST) measures derived from the NASA VIIRS Collection 2 VNP21A1D daytime LST product [25]. Residential locations will be linked to neighborhood-level LST exposures using daily VIIRS observations available from 2012 onward. For residential locations recorded before 2012, comparable daytime LST measures will be derived from the MODIS Aqua product to provide a consistent historical exposure record across the study period [26]. Since individualized daily exposure extraction is not computationally feasible in HRS, LST exposure will be summarized using standardized aggregation windows, including monthly mean LST, annual mean LST, and rolling multi-year mean exposures. Standardized LST z-scores will be calculated by subtracting the core-based statistical area (CBSA)-level mean LST from the participant’s Census tract-level mean LST and dividing by the CBSA-level standard deviation, with higher scores indicating relatively hotter neighborhoods. A secondary heat exposure measure will be the number of extreme heat days, derived from the CDC National Environmental Public Health Tracking Network and linked to participants’ Census tracts over the same exposure windows.

#### Neighborhood and contextual measures.

Neighborhood socioeconomic context (i.e., ADI) [43] and neighborhood tree canopy coverage will be derived using methods parallel to those used in HBI. Ambient fine particulate matter (PM_2.5_) exposure in HRS will be obtained from the restricted-use EPOCH individual-level PM_2.5_ dataset, which provides address-based, time-weighted 1-, 5-, and 10-year average PM_2.5_ concentrations generated from a national spatiotemporal prediction model. To maximize comparability with HBI, primary HRS analyses will use the 1-year and 5-year PM_2.5_ measures. Unlike HBI, these HRS PM_2.5_ estimates are not derived from local ArcGIS interpolation but are provided as an existing address-level exposure product [73,74].

#### Potential mediators.

Social isolation will be assessed using the HRS 6-item social isolation scale, which includes marital status, living arrangement, monthly contact with children, monthly contact with other family members, monthly contact with friends, and participation in groups, clubs, or organizations [75]. Inflammation will be indexed using high-sensitivity C-reactive protein (hs-CRP) among participants included in the 2016 HRS venous blood collection and assay protocol [76]. Physical activity will be summarized using a Physical Activity Index Score derived from self-reported light, moderate, and vigorous activity frequency items, modeled to parallel the HBI physical activity construct [65,77,78].

#### Cognitive assessment.

Cognitive outcomes in the HRS validation sample will be derived from the 2006 wave onward. Global cognition will be assessed using the Telephone Interview for Cognitive Status- modified version (TICSm) [79], a telephone-administered screening measure with scores ranging from 0 to 27 (higher scores indicating better function). It consists of an immediate and delayed word recall task (10 nouns), a backwards counting task from 20−0, and a serial 7s subtraction task over five trials. The total immediate and delayed recall from the word list will be used separately as the memory composite. To address item-level missingness in cognitive data, analyses will use the HRS-released imputed cognition files. Details of the imputation method used have been previously described [80].

#### Covariates.

HRS analyses will adjust for harmonized covariates available in both HBI and HRS and considered potential confounders, including age, sex, individual socioeconomic status, racial group, Hispanic ethnicity, marital status, relevant medical comorbidities where available, neighborhood socioeconomic status, and PM_2.5_ exposure. Additional environmental covariates related to heat exposure, including seasonality and contextual neighborhood characteristics, will be incorporated where available and appropriate.

### Primary and secondary outcomes

The primary and secondary outcomes were selected to evaluate cognitive and biological correlates of neighborhood-level UHI exposure, longitudinal markers of ADRD risk, and potential mechanisms and mitigation pathways across the HBI cohort, the HBI smartwatch sub-study, and the HRS external validation sample.

#### Aim 1.

The primary cross-sectional cognitive outcome in HBI will be executive function, operationalized using the HBI executive-related cognitive factor score. In the smartwatch sub-study, the primary cross-sectional cognitive outcome is daily n-back performance during the baseline monitoring period (S_1_), with reaction time examined secondarily. In HRS, the corresponding validation outcome is the baseline global cognition measured by the modified Telephone Interview for Cognitive Status (TICSm).

#### Aim 2.

The primary longitudinal cognitive outcome in HBI will be the change in global cognition, indexed by the latent general cognitive factor (g), and the key secondary cognitive outcome will be change in episodic memory across annual follow-up visits. Comparable longitudinal cognitive outcomes in HRS are change in global cognition measured by TICSm and change in episodic memory based on word-list recall measures. Primary HBI biomarker outcomes for Aim 2 include hippocampal volume, white matter hyperintensity volume, total cortical volume in AD-relevant regions of interest, and preclinical Alzheimer’s disease risk indexed by APS2 among cognitively unimpaired participants.

#### Aim 3.

The primary outcomes will be the same ADRD-related cognitive and biomarker outcomes examined in Aim 2, used here to evaluate potential mediating and mitigating pathways. These include longitudinal change in global cognition, change in episodic memory, hippocampal volume, white matter hyperintensity volume, total cortical volume in AD-relevant regions, and APS2. The primary mediator variables are physical activity, social isolation, and inflammation. The primary mitigation exposure is neighborhood tree canopy, with UHI exposure evaluated as a mediator of associations between tree canopy and ADRD-related outcomes. In HRS, Aim 3 analyses will be limited to the cognitive outcomes.

### Descriptive analyses

For all aims, we will describe participant demographic and health characteristics, urban heat exposures, cognitive and MRI outcomes, mediators (e.g., physical activity), and the mitigation factor (tree canopy) using descriptive statistics, including means and standard deviations.

### Statistical analyses for Aim 1

Aim 1 will investigate whether recent UHI exposure is associated with concurrent cognitive function. We hypothesize that greater UHI exposure will be cross-sectionally associated with poorer cognitive function (i.e., can cause transient changes in cognition).

#### HBI overall sample.

Linear mixed-effects models will test the cross-sectional association between neighborhood UHI exposure measured at baseline (T), operationalized as the mean LST z-score during the 30 days prior to T, and the executive function factor score measured at baseline. Adjusted models will account for clustering by census tract and will control for age, sex, racial group, Hispanic ethnicity, marital status, Hollingshead Index of Social Position, baseline cognitive status, neighborhood socioeconomic status, and season. Adjusted models will then be re-run controlling additionally for 30-day PM_2.5_ concentrations and comorbidities at T, including cardiovascular disease, cerebrovascular disease, and diabetes.

#### HBI smartwatch sub-sample.

Linear mixed-effects models similar to those above will be used to examine cross-sectional associations between mean daily minutes exposed to UHIs and daily n-back working memory scores during the first smartwatch monitoring period (S_1_). Repeated daily measures of heat exposure and n-back performance will be nested within participants, and models will therefore account for clustering by participant. Only days with both exposure and outcome data available will contribute to the analysis, and models will control for the number of smartwatch days with complete data. Missingness of smartwatch data will be evaluated to determine whether multiple imputation or inverse probability weighting is warranted.

#### HRS validation sample.

Similar linear mixed-effects models will be used to examine cross-sectional associations between neighborhood UHI exposure, indexed by mean LST during the prior 30 days, and baseline global cognition at the baseline HRS cognitive visit.

### Statistical analyses for Aim 2

Aim 2 will determine whether cumulative UHI exposure is associated with plasma AD biomarkers, structural MRI biomarkers, and longitudinal cognitive decline. We hypothesize that greater cumulative urban heat exposure will be associated with steeper decline in global cognition and episodic memory, preclinical AD, lower hippocampal and total cortical AD ROI volumes, and higher WMH volumes.

#### HBI overall sample.

Regression models will be run separately for each exposure-outcome combination. Linear mixed-effects models will test associations between cumulative neighborhood UHI exposure, operationalized as mean LST z-score during the 1- and 5-year periods preceding baseline (T), and the MRI outcomes of hippocampal volume, white matter hyperintensity volume, and total cortical gray matter volume in AD-relevant regions. Adjusted models will account for clustering by census tract and participant and will control for age, sex, racial group, Hispanic ethnicity, marital status, Hollingshead Index of Social Position, baseline cognitive status, cumulative neighborhood SES, and season. These models will then be re-run controlling additionally for cumulative PM_2.5_ and baseline comorbidities, including cardiovascular disease, cerebrovascular disease, and diabetes. Restricting to cognitively unimpaired participants, similar models will test associations between cumulative neighborhood UHI exposure and preclinical AD, (i.e., APS2 [≥47.5]), using logistic regression and without adjustment for cognitive status. Similar linear mixed-effects models will be repeated to test associations between cumulative neighborhood UHI exposure and change in global cognition and change in episodic memory factor score from baseline and subsequent follow-up visits. These models will additionally control for years since baseline and will account for repeated cognitive assessments within participants. Interaction terms between the LST variable and time (e.g., LST1-year × time) will be included to determine whether cumulative urban heat exposure is associated with longitudinal change in cognition.

#### HBI smartwatch sub-sample.

Linear mixed-effects models similar to those conducted for the HBI overall sample will be used to examine longitudinal associations between cumulative mean daily minutes exposed to UHIs, averaged across the two smartwatch monitoring periods S_1_ and S_2_, and the outcomes of global cognition (g), episodic memory, MRI outcomes, and plasma AD biomarker outcomes. Only smartwatch days with UHI exposure data will contribute to the models, and each model will control for the number of smartwatch days with complete data. To preserve temporality, only outcome data collected after the second smartwatch monitoring period (S_2_) will be included. Missing smartwatch data will be evaluated to determine whether multiple imputation or inverse probability weighting is appropriate.

#### HRS validation sample.

Similar linear mixed-effects models from the HBI overall sample analyses will be repeated using HRS data to examine associations between cumulative neighborhood UHI exposure, indexed by mean LST during the 1- and 5-year periods prior to the baseline cognitive visit, and longitudinal change in global cognition and episodic memory.

### Statistical analyses for Aim 3

Aim 3 will evaluate whether (i) UHI exposures are associated with ADRD risk factors (e.g., physical inactivity, inflammation), and (ii) living in neighborhood with more tree canopy is associated with lower urban heat exposures and better ADRD outcomes (e.g., slower cognitive decline) (Mechanisms and Mitigation). We hypothesize that cumulative UHI exposure is associated with physical inactivity, social isolation, greater inflammation, factors that partially mediate heat-ADRD outcome associations. Greater neighborhood tree canopy is associated with less urban heat exposure and better ADRD outcomes, suggesting tree canopy may mitigate urban heat exposure, thereby reducing ADRD risk.

#### HBI analyses of mediation of heat-ADRD associations.

Causal mediation analyses will determine direct and indirect effects between cumulative urban heat exposure, defined as neighborhood mean LST z-scores for the 1- and 5-year periods prior to baseline (T) and mean minutes exposed from smartwatches over S_1_ and S_2_, and the cognitive and biomarker outcomes, including longitudinal change in global cognition, change in episodic memory, hippocampal volume, white matter hyperintensity volume, total cortical volume in AD-relevant regions, and continuous APS2 score. These models will also estimate the extent to which physical activity, social isolation, and inflammation partially mediate those associations. UHI exposure measures will temporally precede the mediators, which will temporally precede the outcomes. Causal mediation linear mixed-effects regression models will be run using the CMAverse package in R, which allows simultaneous consideration of multiple mediators and estimates effects using four-way decomposition: (1) the effect of exposure in the absence of the mediator, (2) the interactive effect when the mediator is present in the absence of exposure, (3) the mediated interaction, and (4) the pure mediated effect. Covariates will include age at baseline, sex, racial group, Hispanic ethnicity, marital status, Hollingshead Index of Social Position, baseline cognitive status (cognitively unimpaired vs. mild cognitive impairment), cumulative neighborhood SES, and season. Sensitivity analyses will additionally control for cumulative PM_2.5_ and comorbidities measured at T or S_1_, depending on the exposure window.

#### HBI analyses of tree canopy as a mitigation factor.

Multivariable causal mediation models similar to those used in Aim 2 models for the HBI overall sample will be run to determine the direct and indirect effects between neighborhood percent tree canopy (at T and S_1_) and the cognitive and biomarker outcomes, including longitudinal change in global cognition, change in episodic memory, hippocampal volume, white matter hyperintensity volume, total cortical volume in AD-relevant regions, and APS2. Mediation models will also determine the extent to which UHI exposure, defined as neighborhood mean LST z-scores from the baseline visit year (T) and mean minutes exposed from smartwatches during S_1_ partially mediates those associations (e.g., greater tree canopy → less UHI exposure → better cognitive and biomarker outcomes). The tree canopy and urban heat measures will temporally precede the outcomes.

#### HRS validation sample.

Regression models similar to those performed for the HBI sample will be repeated using the HRS sample, but limited to the cognitive outcomes as MRI and smartwatch data are not available in HRS.

### Power/minimum detectable effect size (MDES)

Based on projected sample sizes for Aims 1 and 2, we determined the MDES (minimum detectable effect size, f^2^ = R^2^/(1-R^2^), where R^2^ is coefficient of determination) based on assumptions of type I error rate of 5%, 80% power, and inclusion of 8–10 covariates in the regression models (depending on aim). Analyses including all HBI participants (n = 500) will be powered to detect small effect sizes, and HBI smartwatch analyses will be powered to detect medium effect sizes. Aim 2 analyses focused on preclinical AD have >80% power to detect a small effect size. For Aim 3, MDES (β) was calculated assuming type I error rate of 5%, 80% power, and the sample size for the sub-aims, and we determined we will have sufficient power to detect small indirect, direct, and total effects in the mediation models. The HRS validation sample has > 80% power to detect Aims 1–3 associations with n = 9,673 and similar assumptions described above.

### Additional analytic considerations

#### Sex as a biological variable.

Prior research suggests that women may be at an increased risk to extreme heat and ADRD risk differs by sex as such sex differences will be examined in Aims 1–3 using interaction terms (e.g., LST z-score × sex) in multivariable models.

#### Additional analytic considerations.

Multivariable models will be refined as needed to account for additional confounders identified during analysis, as well as non-linearity, non-normality, and/or spatial autocorrelation in the data. When appropriate, variable transformations or alternative model specifications will be considered. To account for multiple comparisons, the Benjamini-Hochberg procedure will be used to control the false discovery rate.

#### Attrition and retention.

For longitudinal analyses, we will compare characteristics of participants who complete follow-up assessments versus those lost to follow-up to evaluate potential attrition bias. If differential attrition is observed, we will use inverse probability of attrition weighting in sensitivity analyses.

### Data management and quality assurance

All study data will be stored and managed securely in accordance with HIPAA requirements, University of Miami data security policies, and applicable data use agreements. Electronic study data, including consent documentation, EMA responses, mobile cognitive task data, and participant contact information for the smartwatch sub-study, will be maintained in secure, access-restricted institutional data systems. Passive smartwatch sensor data, including GPS, accelerometer, and heart rate data, will be stored in HIPAA-compliant encrypted cloud environments. Geospatial datasets, environmental exposure variables, and GIS-derived measures will be stored in secure institutional computing environments with access limited to authorized study personnel using role-based permissions and multi-factor authentication where applicable.

Paper records, including signed consent forms and study documents, will be stored in locked file cabinets within locked research offices with restricted access. Identifiable information, including names, addresses, and contact details, will be stored separately from analytic research data and linked only through coded study identification numbers. The linkage file connecting study IDs to identifiable information will be maintained in a password-protected file accessible only to essential study personnel. All research staff with access to identifiable information or protected health information will complete required human subjects protection and privacy training.

### HRS restricted data access

Access to restricted HRS data containing geocoded residential information will be obtained through a formal Restricted Data Use Agreement with HRS. Analyses involving restricted HRS data will be conducted within the secure HRS Virtual Desktop Infrastructure environment, and identifiable restricted data will not be downloaded to local computers. All HRS data use will comply with the terms of the applicable data use agreement and HRS data security requirements.

### Data quality assurance

Data quality will be monitored throughout the study to promote completeness, accuracy, and consistency across data sources. Participant addresses will be geocoded using standardized procedures, with automated results reviewed for plausibility and manually checked when needed. Neuropsychological data collected in HBI will undergo routine quality assurance procedures to verify scoring accuracy and protocol adherence. MRI-derived measures generated using the Combinostics cNeuro cMRI pipeline will undergo study-level quality checks to identify missing outputs, implausible values, or processing irregularities prior to analysis. Smartwatch-derived data will be reviewed regularly for wear-time completeness, irregular sampling, and GPS quality, and participants may be contacted during monitoring periods if extended non-wear or data capture problems are identified. Biomarker assay data will be reviewed for batch-level quality control, including evaluation of assay performance and reanalysis of samples with values falling outside acceptable ranges. Geospatial exposure measures will also be reviewed for completeness and consistency, and when satellite-derived data are limited because of cloud cover or similar constraints, alternative remote sensing sources and imputation methods will be used as needed.

### Data availability statement

This manuscript describes a study protocol; no study-specific analytic dataset underlying reported findings has yet been generated. Following completion of primary analyses and publication of the main findings, de-identified HEAT-AD derived data that can be shared in accordance with participant consent, institutional policies, and applicable data use agreements will be deposited in a publicly accessible repository, such as the Open Science Framework, with a persistent identifier. Derived geospatial exposure variables created for this project, including urban heat island exposure and tree canopy measures, associated metadata/codebooks, and analysis code will be shared when legally and ethically permissible.

Individual-level HBI data that contain protected health information or potentially identifiable geospatial information will not be publicly posted. Qualified researchers may request access to de-identified or limited HBI datasets from the University of Miami Comprehensive Center for Brain Health, subject to applicable institutional approvals, IRB requirements, and data use agreements. Restricted HRS data, including geocoded residential data, must be requested directly from the Health and Retirement Study through its established restricted data access procedures. Data from participants who withdraw consent for future data use will not be shared after the date of withdrawal.

### Ethical considerations

All study procedures will be conducted in accordance with the ethical principles of the Declaration of Helsinki and the Belmont Report. All research personnel have completed required human subjects protection training, and team members with access to personally identifiable information or protected health information have completed additional privacy and data security training. Analyses involving restricted HRS data will be conducted in compliance with the applicable data use agreement and HRS data security requirements.

### Risk-benefit assessment

This study is considered minimal risk. Potential risks include minor discomfort related to smartwatch wear, modest time burden associated with completion of daily ecological momentary assessment items and mobile cognitive tasks, and a remote risk of breach of confidentiality despite established data security protections. There is also a possibility of mild psychological discomfort related to cognitive testing or other research assessments. These risks will be minimized through monitoring of participant comfort during smartwatch monitoring periods and use of secure data storage systems with encryption, restricted access, and other institutional safeguards.

Participants are unlikely to receive direct individual benefit from participation. However, the study may contribute to knowledge about the relationship between heat exposure and cognitive aging and help inform future public health, environmental, and urban planning interventions.

### Protocol amendments and study termination

Any protocol modifications will be submitted to the Institutional Review Board for review and approval before implementation, as required. Administrative updates will be documented in accordance with IRB policy, and substantive changes affecting study procedures, risk, or participant protections will undergo appropriate review. Relevant protocol amendments will be reflected in study dissemination where applicable.

Early study termination is not expected due to minimal risk. Any decision regarding early termination will be made in consultation with the IRB and, where applicable, the funding agency. In the event of early termination, ongoing data collection will cease, participants will be notified as appropriate, and existing data will be retained, secured, and handled in accordance with approved ethical and regulatory procedures.

### Study status and timeline

The parent HBI cohort study is ongoing. The HEAT-AD project funding began in September 2025 and ends on April 30, 2030. The HEAT-AD study has not yet generated results from the planned analyses described in this protocol. Start-up activities, including staff training, smartwatch programming, institutional review board approvals, and data use agreement procedures, began during the first project year. Recruitment for the HEAT-AD smartwatch sub-study began on April 30, 2026, and the first smartwatch sub-study participant provided informed consent on May 5, 2026. Recruitment and data collection for the smartwatch sub-study are ongoing and are expected to continue through March 30, 2030. Environmental data assembly and GIS-based exposure derivation will occur throughout the project period, with major exposure derivation activities expected during 2026–2028. Processing of smartwatch-derived variables, quality control procedures, and harmonization with HBI and HRS data will occur alongside data collection and continue through 2030. Preliminary analyses are expected to begin in 2030 after completion of the relevant exposure derivation and follow-up data processing. Primary study results are expected by late 2030.

## Discussion

### Study summary

The HEAT-AD study is a multi-cohort observational study examining associations between UHI exposure and ADRD risk in older adults. The study integrates two main data sources: the HBI cohort in South Florida (n = 500), including a nested smartwatch sub-study with intensive prospective monitoring (n = 200), and the HRS for national validation (n = 9,673). Participants’ geocoded residential addresses are linked to satellite-derived land surface temperature data to quantify neighborhood-level UHI exposure over multiple time windows. These exposure measures are examined in relation to comprehensive outcomes including neuropsychological assessment, structural MRI, and plasma biomarkers of preclinical Alzheimer’s disease. The smartwatch sub-study provides granular individual-level data on GPS-based heat exposure and mobility patterns during two 3-week monitoring periods, complemented by repeated mobile cognitive testing. The study aims to determine: (1) whether recent UHI exposure is associated with concurrent cognitive function, (2) whether cumulative UHI exposure is associated with longitudinal cognitive decline and biomarkers of neurodegeneration, and (3) whether associations are mediated by physical inactivity, social isolation, and inflammation, and mitigated by neighborhood tree canopy coverage.

### Strengths and innovations

This study has several innovative features that advance the field beyond prior research. First, to our knowledge, it is among the first studies of cumulative UHI exposure rather than acute heat waves or regional air temperatures in relation to ADRD-specific outcomes. By quantifying chronic neighborhood-level heat burden measured via satellite-derived land surface temperatures over multiple years, this study tests whether environmental heat acts as a persistent risk factor for neurodegeneration rather than solely an acute cognitive stressor. UHIs represent a particularly relevant exposure given that they reflect modifiable features of the built environment, are disproportionately prevalent in economically lower resourced neighborhoods, and may be addressable through environmental and public health interventions [2,26,81].

Second, the integration of satellite-derived heat metrics with real-world behavioral data from wearable devices provides an ecologically valid assessment of heat exposure that accounts for individual mobility patterns and adaptive behaviors [82,83]. While satellite imagery alone provides high-resolution estimates of neighborhood heat exposure, these residence-based measures do not capture how individuals interact with their environments. The smartwatch sub-study addresses this limitation by linking continuous GPS trajectories to land surface temperature maps, yielding individualized estimates of time spent in UHI conditions during daily life. This approach enables examination of how neighborhood heat interacts with daily routines, outdoor activities, and avoidance behaviors.

Third, the wearable-based assessment of short-term cognitive vulnerability through repeated mobile cognitive testing is a novel methodological approach. The mobile implementation of the n-back working memory task provides a brief, validated measure that is well suited for frequent administration and sensitive to within-person fluctuations [71]. Embedding cognitive assessment within the same temporal window as wearable-based heat monitoring allows for evaluation of short-term associations between heat exposure and executive functioning, complementing traditional annual neuropsychological assessments and bridging laboratory-based cognitive testing with real-world exposure.

Fourth, this study examines UHI exposure in relation to biological markers of Alzheimer’s disease pathology (plasma biomarkers) and neurodegeneration (structural MRI). Structural brain imaging biomarkers can provide insight into neurodegenerative processes that unfold years before the onset of clinical impairment [61,84]. Similarly, blood-based biomarkers of amyloid pathology (APS2) [62] may help elucidate biological mechanisms through which heat-related stressors influence disease risk at the preclinical stage. To our knowledge, no prior studies have examined UHI exposure in relation to these outcomes.

Fifth, the explicit examination of neighborhood tree canopy as a modifiable environmental feature offers direct evidence relevant to urban planning and intervention strategies. Tree canopy represents a distinct and actionable component of the built environment that can meaningfully reduce surface temperatures and improve outdoor comfort [81,85,86]. By quantifying tree canopy coverage and examining whether it buffers against UHI exposure and improves ADRD outcomes, this study provides empirical evidence that could inform urban greening interventions for ADRD prevention.

Finally, this study adopts a mechanistic, pathway-oriented approach by evaluating physical activity, social isolation, and systemic inflammation as potential mediators linking neighborhood heat exposure to cognitive and brain health outcomes. By integrating self-reported measures, wearable-derived behavioral data, and blood-based inflammatory biomarkers, this study is positioned to assess how environmental heat translates into downstream behavioral and physiological changes. This multidimensional approach advances understanding of how and why UHI exposure may contribute to cognitive aging and ADRD risk, rather than merely documenting associations.

Methodological strengths include the use of HBI, a deeply phenotyped cohort with comprehensive cognitive assessment, the longitudinal study design enabling examination of cumulative exposure and cognitive trajectories, the ethnoracial diversity of the South Florida study population, the external validation in a nationally representative sample (HRS), and the application of rigorous causal inference methods including mediation analysis to examine mechanisms and tree canopy mitigation.

### Limitations and mitigation strategies

This study has several limitations that should be acknowledged. First, the observational design limits causal inference. While we cannot randomly assign participants to different levels of heat exposure, we mitigate this through longitudinal data collection with exposure assessed prior to outcomes to establish temporal precedence, extensive covariates to adjust for potential confounding, sensitivity analyses adjusting for air pollution and comorbidities, causal mediation analysis methods, and external validation in an independent national sample.

Second, the HBI cohort consists of health-conscious volunteers willing to undergo intensive assessments, which may limit generalizability. This limitation is partially offset by deliberate recruitment strategies to achieve ethnoracial diversity (at least 35% participants from minority groups), inclusion of participants across the cognitive spectrum from cognitively unimpaired to mild dementia, recruitment from diverse neighborhoods, and external validation in the nationally representative HRS.

Third, satellite-derived land surface temperature measures outdoor surface temperatures and may not fully capture indoor heat exposure, which depends on housing characteristics, air conditioning access, and individual behaviors. The smartwatch sub-study helps address this by providing individual-level, GPS-based assessment of actual locations visited and time spent in UHI environments during daily activities.

Fourth, the smartwatch sub-study sample size (n = 200) may be underpowered for some subgroup analyses or to detect small effect sizes. To mitigate this, we will enroll up to 225 participants to account for attrition and will focus smartwatch analyses on detection of medium effect sizes, which are adequately powered and most likely to be clinically meaningful. Borderline associations due to limited statistical power can be explored in future studies with larger samples.

Fifth, satellite imagery can be affected by cloud cover, potentially resulting in missing land surface temperature data. We will address this by using multiple satellite data sources as needed (VIIRS, MODIS) to maximize temporal coverage, applying temporal interpolation methods when appropriate, and documenting the extent of missing data and conducting sensitivity analyses.

Sixth, retention in longitudinal studies is always a challenge. We will support retention of the HBI participants through regular communication, flexible visit scheduling, provision of study results in accessible formats, compensation for time, and maintaining strong relationships with participants and study partners. For smartwatch participants who miss HBI annual visits, we will make additional outreach efforts to recruit them for the smartwatch sessions.

### Anticipated contributions and significance

This study will provide novel evidence on whether chronic exposure to UHI environments represents a modifiable risk factor for ADRD. By mid-century, extreme heat exposure is projected to increase several-fold [5], while the number of Americans living with ADRD is expected to double [1]. Understanding the intersection of these two major public health challenges is essential for developing effective prevention strategies.

If UHI exposure is associated with cognitive decline, structural brain changes, or preclinical Alzheimer’s pathology, this would establish environmental heat as a potential target for population-level ADRD prevention efforts. Importantly, unlike individual-level risk factors such as diet or exercise, environmental interventions targeting the built environment such as increasing tree canopy coverage, creating green spaces, and improving urban design can provide sustained benefits to entire communities without requiring ongoing individual behavior change. Such interventions may be particularly impactful in neighborhoods with fewer resources that currently experience the highest UHI effects and bear disproportionate ADRD burden [2,26].

If mediating pathways through physical activity, social isolation, or inflammation are identified, this would clarify biological and behavioral mechanisms and could inform multi-level intervention approaches. For example, if physical inactivity mediates heat-ADRD associations, interventions could target both environmental cooling (to reduce heat exposure) and promotion of physical activity in climate-controlled environments.

Methodologically, this study establishes approaches for integrating satellite-derived environmental exposure data with comprehensive neuropsychological, neuroimaging, and biomarker phenotyping. The methods developed here are extensible to other environmental exposures (e.g., air pollution, greenspace, noise) and other health outcomes (e.g., cardiovascular disease, mental health). The smartwatch-based approach to measuring individual-level environmental exposure and ecological momentary cognitive assessment represents an innovative method that could be adopted for future environmental health research.

### Conclusion

The HEAT-AD study represents a rigorous investigation of UHI exposure and Alzheimer’s disease risk in a diverse cohort of older adults. This study comprehensively examines cumulative UHI exposure, ADRD biomarkers, and mediating pathways. Findings will provide evidence on whether neighborhood heat environments represent a modifiable target for ADRD prevention. In the context of accelerating extreme heat events and population aging, understanding environmental determinants of cognitive health has never been more urgent. The findings from this study have the potential to inform urban planning, environmental policy, and public health strategies aimed at promoting brain health and reducing health disparities in aging populations.
